# Prevalence of human papillomavirus in head and neck cancer patients in India: a systematic review and meta-analysis

**DOI:** 10.1186/s12879-024-09357-2

**Published:** 2024-05-23

**Authors:** Prakasini Satapathy, Mahalaqua Nazli Khatib, Shilpa Gaidhane, Quazi Syed Zahiruddin, Hashem Abu Serhan, Rakesh Kumar Sharma, Divya Sharma, Mithhil Arora, Sarvesh Rustagi, AlKaabi NA, Ahmed Alsayyah, Marwan Al-Hajeili, Maha F. Al-Subaie, Mubarak Alfaresi, Mohammed Alissa, Ali A. Rabaan

**Affiliations:** 1 Center for Global Health Research, Saveetha Institute of Medical and Technical Sciences, Saveetha Medical College and Hospital, Saveetha University, Chennai, India; 2 Medical Laboratories Techniques Department, AL-Mustaqbal University, Hillah, Babil, 51001 Iraq; 3 Division of Evidence Synthesis, Global Consortium of Public Health and Research, Datta Meghe Institute of Higher Education, Wardha, India; 4 One Health Centre (COHERD), Jawaharlal Nehru Medical College, Datta Meghe Institute of Higher Education, Wardha, India; 5 Global South Asia Infant Feeding Research Network (SAIFRN), Division of Evidence Synthesis, Global Consortium of Public Health and Research, Datta Meghe Institute of Higher Education, Wardha, India; 6 Department of Ophthalmology, Hamad Medical Corporation, Doha, Qatar; 7 Graphic Era (Deemed to be University), Clement Town, Dehradun, India; 8 Graphic Era Hill University, Clement Town, Dehradun, India; 9 Centre of Research Impact and Outcome, Chitkara University, Rajpura, 140417 Punjab India; 10 Chitkara Centre for Research and Development, Chitkara University, Himachal Pradesh, 174103 India; 11 School of Applied and Life Sciences, Uttaranchal University, Dehradun, Uttarakhand India; 12 College of Medicine and Health Science, Khalifa University, 127788 Abu Dhabi, United Arab Emirates; 13 Sheikh Khalifa Medical City, Abu Dhabi Health Services Company (SEHA), 51900 Abu Dhabi, United Arab Emirates; 14 Department of Pathology, College of Medicine, Imam Abdulrahman Bin Faisal University, 31441 Dammam, Saudi Arabia; 15 Department of Medicine, College of Medicine, King Abdulaziz University, 23624 Jeddah, Saudi Arabia; 16 Research Center, Dr. Sulaiman Alhabib Medical Group, 13328 Riyadh, Saudi Arabia; 17 College of Medicine, Alfaisal University, 11533 Riyadh, Saudi Arabia; 18 Department of Microbiology, National Reference laboratory, Cleveland clinic Abu Dhabi, 92323 Abu Dhabi, United Arab Emirates; 19 Department of Pathology, College of Medicine, Mohammed Bin Rashid University of Medicine and Health Sciences, 505055 Dubai, United Arab Emirates; 20 Department of Medical Laboratory, College of Applied Medical Sciences, Prince Sattam bin Abdulaziz University, 11942 Al-Kharj, Saudi Arabia; 21 Molecular Diagnostic Laboratory, Johns Hopkins Aramco Healthcare, 31311 Dhahran, Saudi Arabia; 22 Department of Public Health and Nutrition, The University of Haripur, 22610 Haripur, Pakistan

**Keywords:** HPV, Head and neck cancers, Prevalence, India, Vaccination, Public health interventions, Epidemiology

## Abstract

**Background:**

Human papillomavirus (HPV) is increasingly recognized as a significant risk factor in the development of head and neck cancers (HNCs), with varying prevalence and impact. This study aims to systematically review and analyze the prevalence of HPV in HNCs in India, providing insights into regional variations.

**Methods:**

A comprehensive literature search was carried out using PubMed, Embase, and Web of Science up to November 10, 2023. Inclusion criteria focused on original research reporting HPV-positive cases among HNC patients in India. We used Nested-Knowledge software, for screening, and data extraction. The modified Newcastle-Ottawa Scale was used for quality assessment of included studies. We pooled the prevalence of HPV among HNC patients and performed a random-effects model meta-analysis using R software (version 4.3).

**Results:**

The search yielded 33 studies, encompassing 4654 HNC patients. The pooled prevalence of HPV infection was found to be 33% (95% CI: 25.8–42.6), with notable heterogeneity (I² = 95%). Analysis of subgroups according to geographical location indicated varying prevalence rates. Specifically, the prevalence was 47% (95% CI: 32.2–62.4) in the eastern regions and 19.8% (95% CI: 10.8–33.4) in the western regions. No evidence of publication bias was detected.

**Conclusion:**

The observed considerable regional disparities on the prevalence of HPV in HNC patients in India emphasizes the need for integrated HPV vaccination and screening programs in public health strategies. The findings underline the necessity for further research to explore regional variations and treatment responses in HPV-associated HNCs, considering the impact of factors such as tobacco use and the potential benefits of HPV vaccination.

**Supplementary Information:**

The online version contains supplementary material available at 10.1186/s12879-024-09357-2.

## Introduction

Head and neck cancers (HNC), also known as head and neck squamous cell carcinomas (HNSCC), consist of various malignancies impacting the mucosal surfaces of the upper aerodigestive tract, in areas like the nasopharynx, oral cavity, larynx, oropharynx, hypopharynx, and paranasal sinuses [1]. Annually, HNSCC is responsible for over 650,000 new cancer cases and causes more than 350,000 deaths worldwide [2–4]. Traditional primary risk factors for these cancers have been alcohol and tobacco use. However, in recent years, the human papillomavirus (HPV) has been recognized as a significant emerging risk factor, particularly for oropharyngeal squamous cell carcinoma (OPSCC). This has led to the identification of a distinct subtype of HPV-related tumors, which differ from those not associated with HPV. The prevalence of HNSCC tends to vary based on the specific anatomical location and the geographic area [5].

The carcinogenic nature of HPV was definitively established in 1983 when Durst et al. successfully cloned HPV type 16 from cervical carcinoma tissue [6]. It is now widely acknowledged that high-risk HPV types are accountable for almost all cases of cervical cancer. While most HPV infections are asymptomatic and tend to resolve spontaneously, persistent HPV infection in the basal cells of the cervix can lead to the development of cervical cancer [6, 7].

Numerous research articles indicate that epithelial cells from the oral cavity and tonsils can undergo immortalization through the influence of the full-length HPV-16 or its E6/E7 oncogenes [8–12]. Furthermore, studies using transgenic mice have shown that the E6/E7 genes of HPV 16 significantly heighten the risk of developing oral and oropharyngeal cancers [13]. It was found that the E7 gene, in particular, is more effective in triggering these cancers, yet there is a noticeable synergistic effect between E6 and E7 in the genesis of HNSCC [14].

In 2020, India accounted for 7% of the total cancer cases worldwide and a significant 24% of the global incidence of HPV-related cancers [15]. Additionally, it was reported that 80% of cervical cancer cases in India were attributed to HPV subtypes 16 and 18 [16, 17]. As part of its cancer control strategy, India has started implementing opportunistic screening programs for common cancers, including those of the cervix and oral cavity, in its healthcare facilities [18]. A notable increase in HNC occurrences was observed in the population-based cancer registries (PBCRs) for cities such as Aurangabad, Delhi, Chennai, and Bhopal among men, and in Nagpur among women, in India [19]. The age-adjusted incidence rate of HNC stood at approximately 25.9 (95% CI 25.7–26.1) and 8.0 (95% CI 7.9–8.1) per 100,000 people for men and women, respectively [20]. HNC represented around 26% of all cancer diagnoses in men and 8% in women. The likelihood of being diagnosed with HNC was 1 in 33 for men and 1 in 107 for women [20].

Despite the growing body of literature on HPV’s role in HNC, there remains a lack of consensus on its prevalence and impact. This variability is partly due to differences in study designs, population demographics, and detection methods used across various research studies. A systematic review and meta-analysis of the existing studies can provide a more comprehensive understanding of HPV’s prevalence in HNC, offering insights into regional variations.

## Method

This systematic review was conducted in accordance with PRISMA guidelines [21] (Table S1) and has been registered in PROSPERO.

### Literature search

An electronic literature search was conducted in various databases, including PubMed, Embase, and Web of Science from inception up to November 10, 2023. Keywords and MeSH terms related to HPV and HNC were used to devise the search strategy. No restrictions have been placed on the type of article, year of publication, or language in the search. Table S2 shows the detailed search strategy.

### Inclusion criteria

Original research that reports the number of HPV-positive cases among HNC patients is included in this study. We are considering only studies conducted in India. Excluded from this study are case reports, case series, or studies lacking quantitative data. The same exclusion criteria apply to animal studies, commentaries, in vitro studies, and reviews. Studies from any location within India, whether conducted in a hospital or community setting, are eligible. There are no restrictions on the type of test used to detect HPV. A detailed inclusion criterion is specified in Table S3.

### Screening and study selection

Two independent reviewers performed the screening of the articles. Nested-Knowledge software was employed for this process. The screening involved a primary review of titles and abstracts, followed by a full-text reading to assess the eligibility of studies for inclusion. An independent third reviewer was consulted to resolve any discrepancies.

### Data extraction and quality assessment

Data extraction was performed using the Tag function of the Nested-Knowledge software. Three reviewers carried out the data extraction process. A fourth reviewer cross-checked and validated the extracted data. The data extracted covered the author’s name, year of publication, state/location of the study, study design, age and percentage of males in the sample, total number of HNC patients in the study, number of HPV-positive samples, and the type of test employed for detecting HPV. Quality assessment was done using a modified Newcastle-Ottawa Scale (NOS) version [22, 23].

### Statistical analysis

A pooled prevalence of HPV is determined by performing a meta-analysis. A random-effects model is employed to conduct the meta-analysis. The variability in study outcomes was measured using the I^2^ statistic, which quantifies heterogeneity on a scale from 0 to 100%, where higher values indicate greater heterogeneity [24]. The heterogeneity was further assessed using the 95% prediction interval. We calculated the tau-squared value using maximum likelihood estimation to gain additional insights into heterogeneity [25–28]. Subgroup analysis was conducted based on the location of the study. We used a funnel plot and the Egger test to detect any potential publication bias. A p-value below 0.05 was typically regarded as statistically significant. All statistical analyses were performed using R software, version 4.3 [23, 29, 30].

## Results

### Literature search

**Fig. 1:**
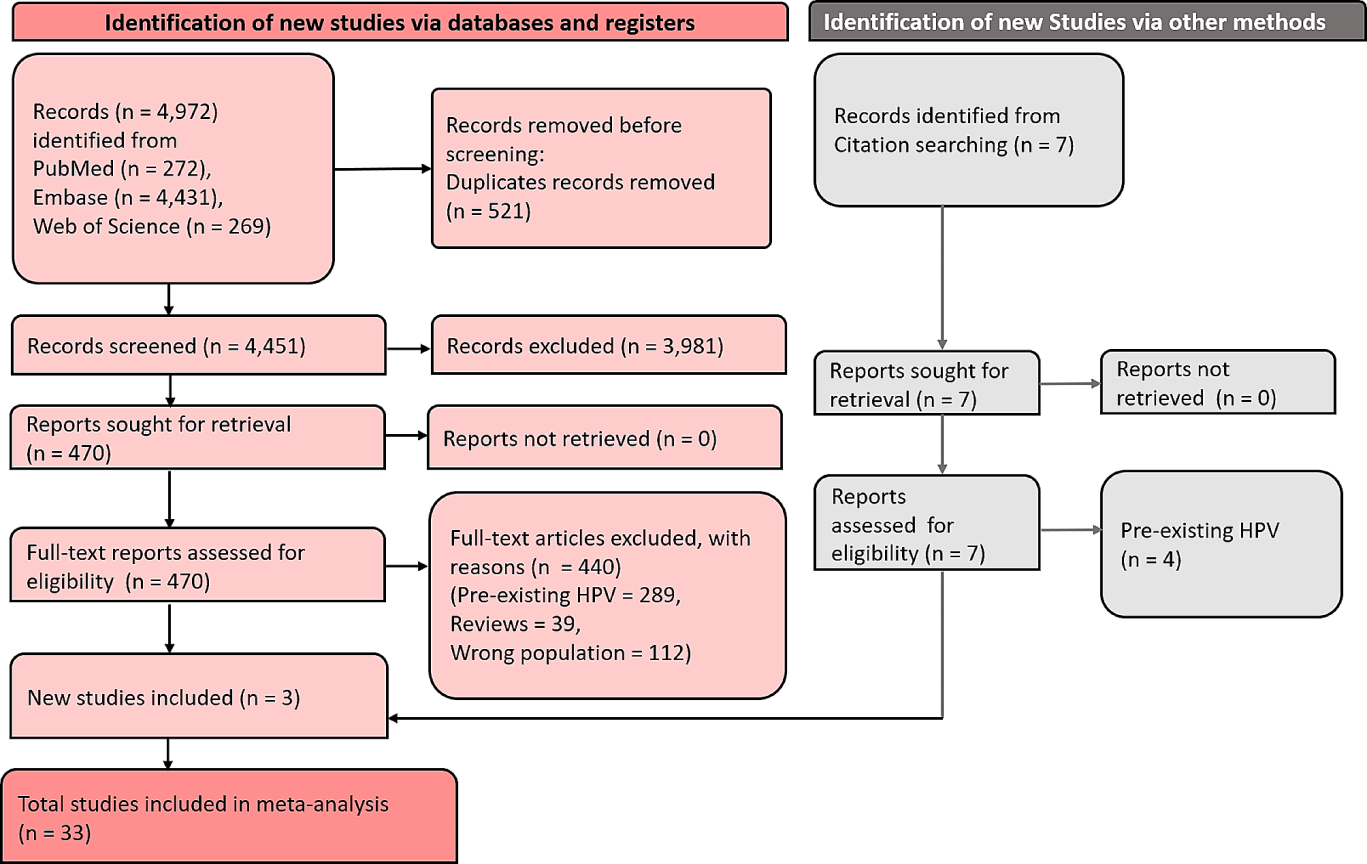
PRISMA flow diagram showing the screening and selection process

In the literature search, 4,972 articles were identified from multiple databases. Among these, 521 were duplicates. After removing duplicates, 4,451 articles were subjected to screening, of which 3,981 were excluded. The remaining 470 articles were screened by full text for eligibility, resulting in the exclusion of 440, leaving 30 for inclusion. Additionally, 3 studies were added from a citation search. Finally, 33 studies were included in the review. Figure 1 depicts the PRISMA flow chart of the selection and screening process.

### Characteristics of included studies

The studies included in this review examining the prevalence of HPV in HNC cases in India are characterized by a diverse methodology and geographical spread. Table 1 summarises the characteristics of included studies. These investigations employ various study designs, including prospective, retrospective, case-control, cohort, and cross-sectional studies, providing a thorough exploration of the subject. The studies are conducted across multiple states and regions of India, representing a broad geographic distribution, and include participants across different age groups, offering insights into age-related variations in HPV prevalence. The proportion of male participants in these studies varies, suggesting potential gender differences in HPV prevalence. The primary diagnostic methods used in these studies are Polymerase Chain Reaction (PCR), genotyping, PCR and P16 Immunohistochemistry (IHC), and Chromogenic In Situ Hybridization (ISH), noted for their high sensitivity. The size of the study populations ranges from small cohorts to large groups, affecting the statistical power of the findings. Rates of HPV positivity exhibit considerable variability, underlining the complexity of understanding HPV prevalence in HNC in this context. These studies span a wide time frame, allowing for the analysis of temporal trends in HPV prevalence. New Delhi contributed 4 studies to the research on HPV prevalence in HNC [31–33], while Uttar Pradesh was represented by 5 studies [34–38]. Maharashtra was the focus of 4 studies [39–42]. Multiple studies were conducted in South India [43, 44], Haryana [45–47], and Tamil Nadu [48, 49]. Single studies were conducted in several other locations: Andhra Pradesh [50], Kerala [51], Kolkata [52], Mumbai [53, 54], Karnataka [55, 56], Chandigarh [57, 58], and Assam [59, 60]. Additionally, one study was a multi-regional study encompassing several states [42]. The included studies were overall of moderate to high quality in modified NOS (Table S4).

**Table 1:** Characteristics of studies

Study	Study design	Age	Male	Type of cancer	No. of Head and neck cancer patients	No. of patients with HPV	State/region	Diagnostic method
Bahl 2014 [31]	Prospective study	55 (Median)	86	OSCC	105	24	New Delhi	Genotyping
Balaram 1995 [51]	Cross sectional study	NA	54.94	Oral cavity cancer	91	67	Kerala	PCR
Barwad 2011 [57]	Prospective study	NA	87.38	Head and neck cancer	111	36	Chandigarh	PCR
Chowdary 2018 [50]	Case-control study	NA	55	Head and neck cancer	20	11	Andhra Pradesh	PCR
Costa 1998 [53]	NA	50 (median)	72.00	Oral cavity cancer	100	15	Mumbai	PCR
Elango 2011 [43]	Cross sectional study	55.00	68.33	Oral or Tongue cancer	60	29	South India	PCR and P16 IHC
Gheit 2017 [39]	Retrospective study	53.6	72.3	NA	364	50	Maharashtra	Genotyping
Gholap 2022 [40]	Cross sectional study	52.26	84.57	Head and neck cancer	175	54	Maharashtra	NA
Jalouli 2010 [34]	Cross-sectional study	OSMF-40.66 ± 15.40;	NA	OSCC	62	15	Uttar Pradesh	PCR
Jitani 2015 [52]	Cross sectional study	52.2 (median)	51.61	Oral cavity cancer	31	9	Kolkata	Chromogenic ISH
Kane 2015 [41]	Retrospective study	43 (median)	87.90	Carcinoma oropharynx	124	16	Maharashtra	NA
Koppikar 2005 [54]	Prospective study	OSCC-58.23 ± 9.6		Squamous cell carcinoma of head and neck	102	32	Mumbai	PCR
Kulkarni 2011 [55]	NA	NA	NA	Oral cavity cancer	34	24	Karnataka	NA
Kumar 2015 [59]	NA	53.41	68.96	Head and neck cancer	106	33	Assam	NMPCR amplified/HC-II assay
Mishra 2006 [32]	Cross sectional study	51	65.7	OSCC	66	18	New Delhi	PCR
Mitra 2007 [82]	NA	52.9	NA	HNSCC	86	59	West Bengal	PCR
Mondal 2013 [60]	NA	58 (median)	79.03	Oral cavity cancer	124	54	Assam	PCR
Murthy 2016 [42]	Cohort study	46.94	84.2	OSCC	170	67	Multiple	PCR
Nagpal 2001 [83]	Cross sectional study	NA	61.81	Oral cavity	110	37	Orissa	PCR
Naz 2022 [33]	Cohort study	54 (Median)	88.8	OSCC	800	104	New Delhi	PCR
Panneerselvam 2019 [44]	Cross sectional study	45.2	87.6	OSCC	30	20	South India	PCR
Parshad 2015 [45]	Prospective study	55.32	88.00	Oral cavity	50	21	Haryana	PCR
Rajesh 2017 [56]	Cross sectional study	NA	90	oral squamous cell carcinoma	60	0	Karnataka	PCR
Ralli 2016 [46]	Prospective study	54.30	85.33	Head and neck cancer	75	59	Haryana	P16 IHC
Ramshankar 2014 [48]	Retrospective study	NA	65.45	Oral and Tongue cancer	167	86	Tamil Nadu	PCR
Sannigrahi 2016 [58]	NA	53.7	25	Head and neck cancer	226	67	Chandigarh	PCR
Sarkar 2017 [84]	NA	NR	86.2	HNSCC	436	278	West Bengal	PCR
Singh 2015 [35]	Prospective study	52	75.9	OSCC	250	23	Uttar Pradesh	PCR
Singh 2016 [36]	Prospective study	NA	80	OSCC	43	3	Uttar Pradesh	PCR
Sivakumar 2021 [47]	Prospective study	45.56	86	OPSCC, OSCC	38	18	Haryana	PCR
Sri 2021 [49]	Cross sectional study	46.96	NA	OSCC	40	8	Tamil Nadu	PCR
Vanshika 2021 [37]	Cross sectional study	NR	NA	Oral cancer	108	14	Uttar Pradesh	PCR
Verma 2017 [38]	Prospective study	51.80	110.00	Oral cavity and oropharynx	135	31	Uttar Pradesh	PCR and P16 IHC

HNSCC: Head and Neck Squamous Cell Carcinoma, IHC: Immunohistochemistry, NMPCR: Nested Multiplex Polymerase Chain Reaction, OSCC: Oral Squamous Cell Carcinoma, PCR: Polymerase Chain Reaction, OPSCC: Oropharyngeal Squamous Cell Carcinoma

### Meta-analysis

**Fig. 2:**
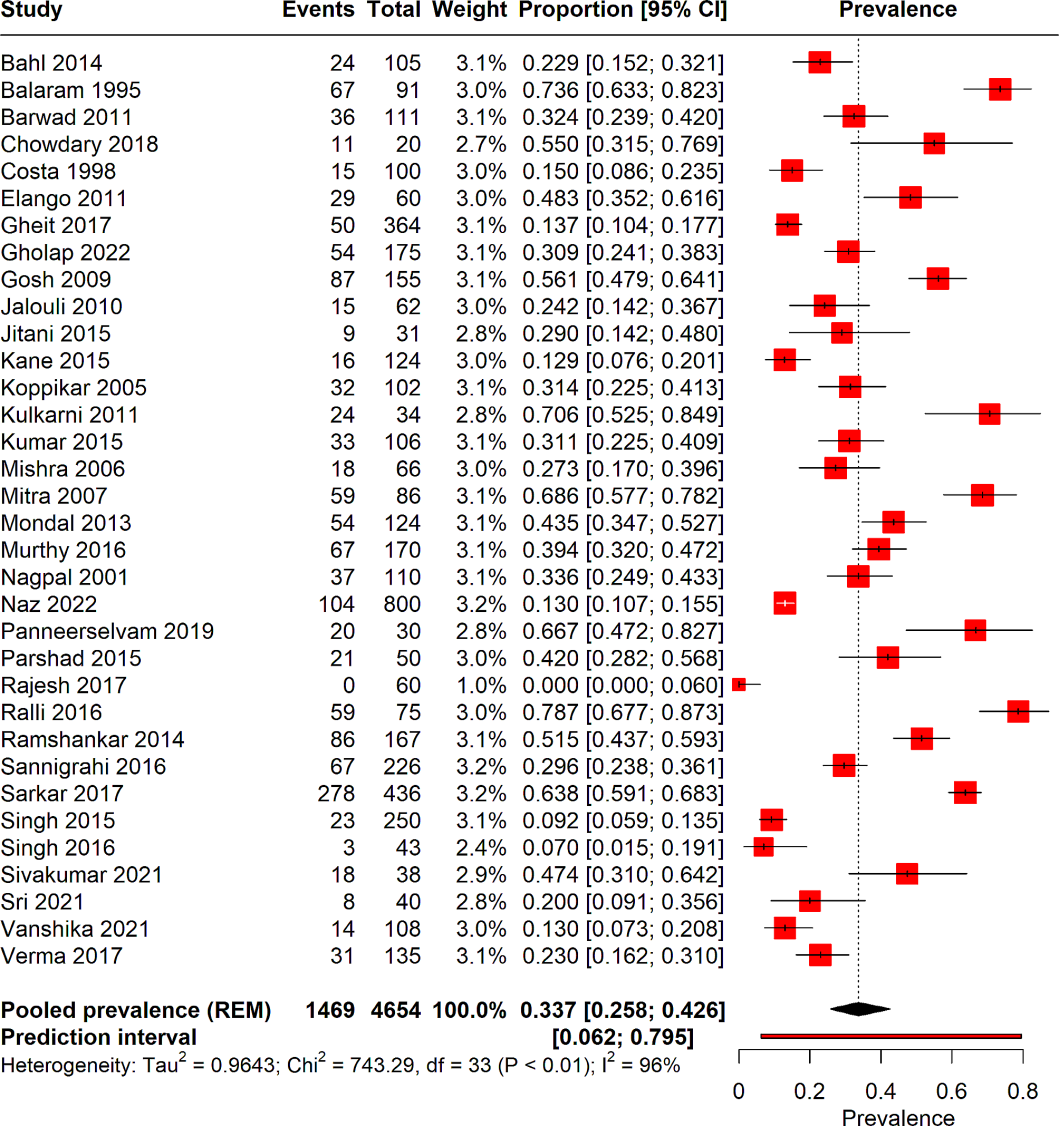
Forest plot depicting the overall HPV pooled prevalence in head and neck cancer patients

From 33 studies encompassing a total of 4654 patients with HNC, the pooled prevalence of HPV infection was determined to be 33% (95% CI: 25–42%). Notably, substantial heterogeneity was observed among these studies (I² = 96%). A prediction interval ranging from 6.2 to 79% was also observed. Figure 2 illustrates the forest plot depicting the pooled prevalence.

### Subgroup analysis

**Fig. 3:**
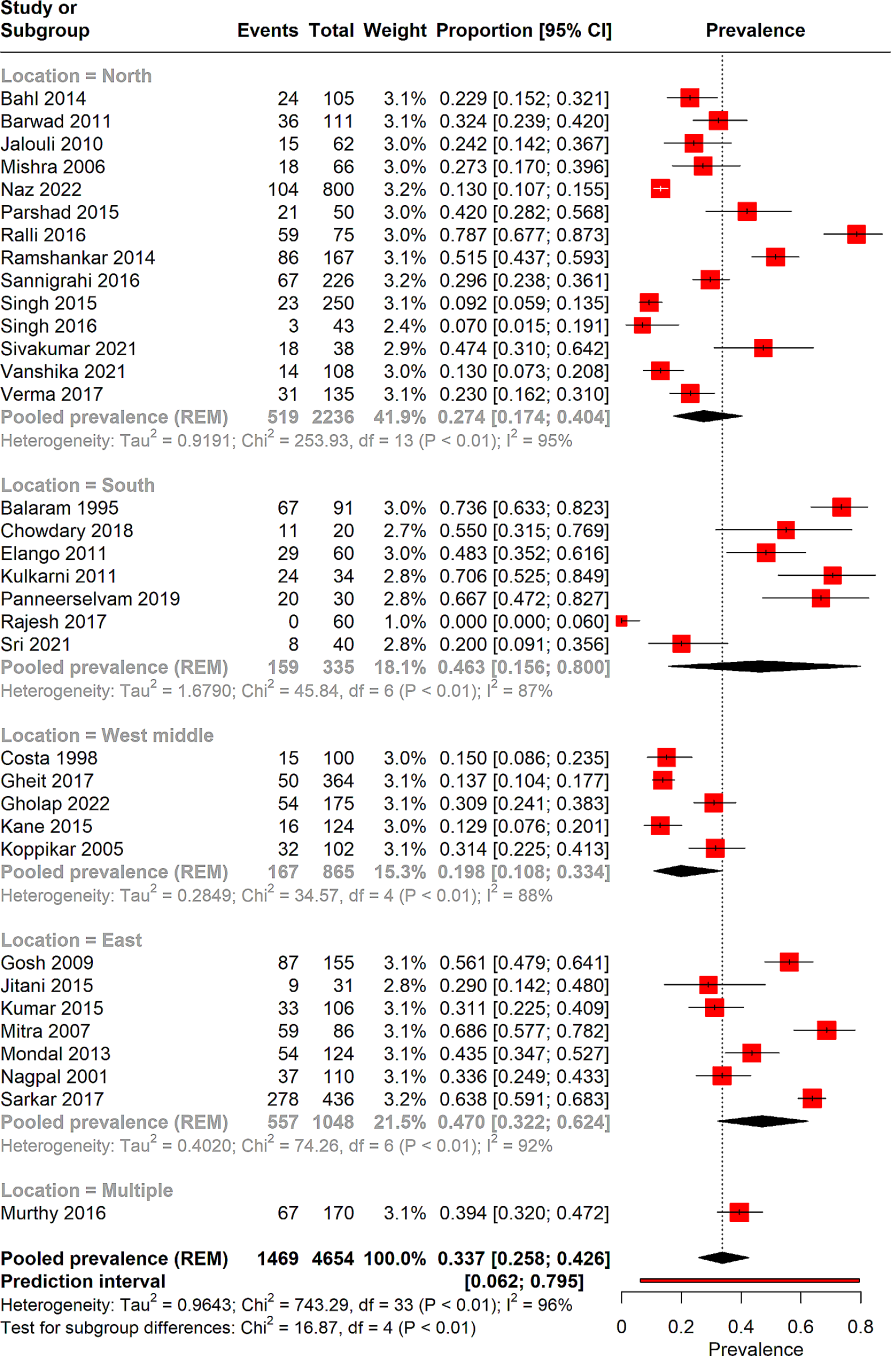
Forest plot illustrating the results of sub-group analysis-based location

Subgroup analysis was performed on the basis of the location of the study (Fig. 3). The North region is represented by 14 studies encompassing a total of 2236 patients, revealing a pooled HPV prevalence of 27% (95% CI: 17.4–40.4%), and a high degree of heterogeneity (I² = 95%). The South region, with 7 studies and 335 patients, has a pooled prevalence of 46.3% (95% CI: 15.6–80%), also accompanied by significant heterogeneity (I² = 87%). In the West Middle region, 5 studies totalling 865 patients show a pooled prevalence of 19.8% (95% CI: 10.8–33.4%) with substantial heterogeneity (I² = 88%). A single study spans multiple locations—Madhya Pradesh, Gujarat, Rajasthan, Uttar Pradesh, West Bengal, and Assam—encompassing 170 patients and reporting a higher pooled prevalence of 39% (95% CI: 32–47%). Lastly, the East region, represented by 7 studies with 1048 patients, presents a pooled prevalence of 47% (95% CI: 32.2–62.4%) with high heterogeneity (I² = 92%).

### Meta-regression and sensitivity analysis

**Fig. 4:**
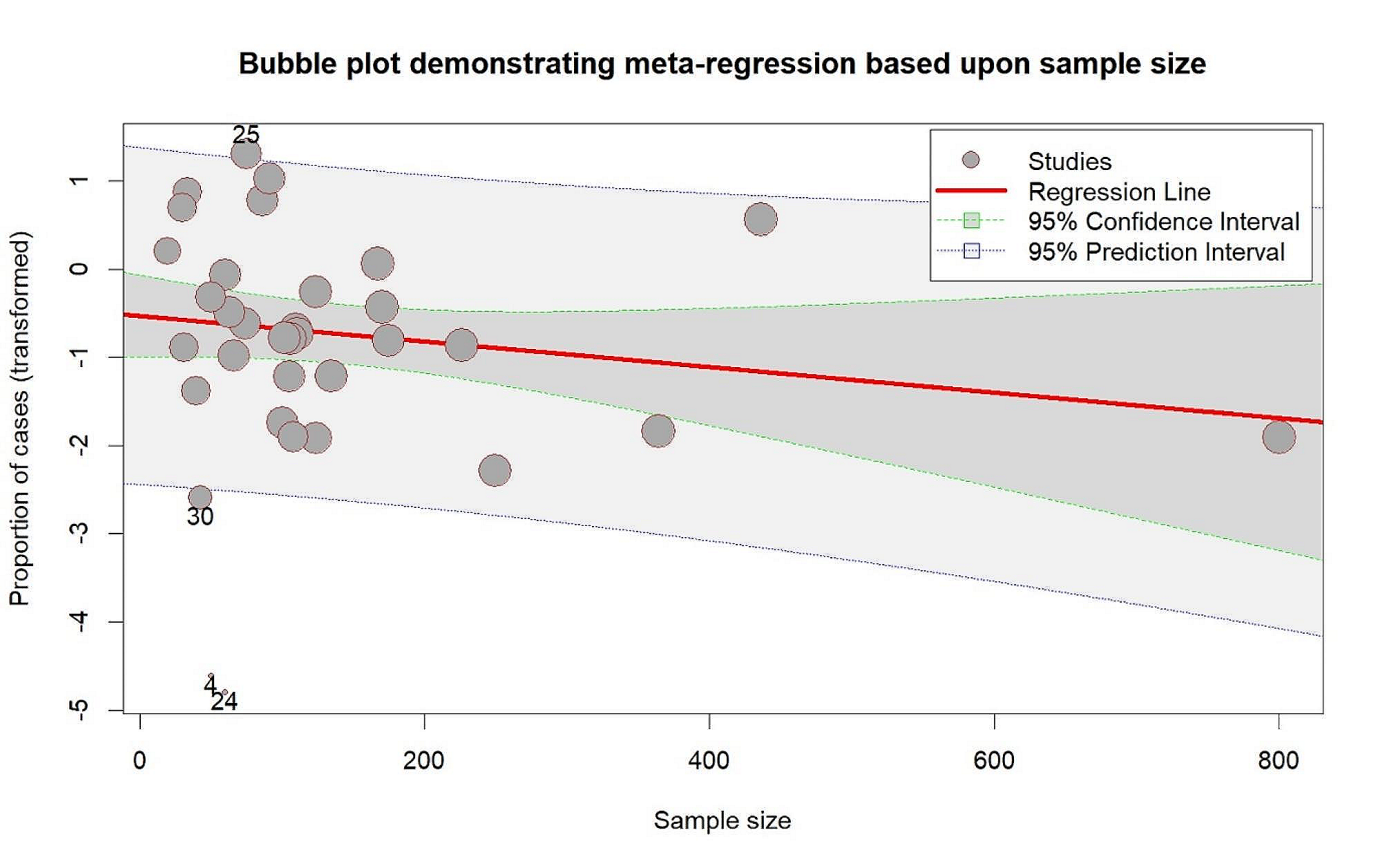
Bubble plot depicting the results of meta-regression based on sample size

**Fig. 5:**
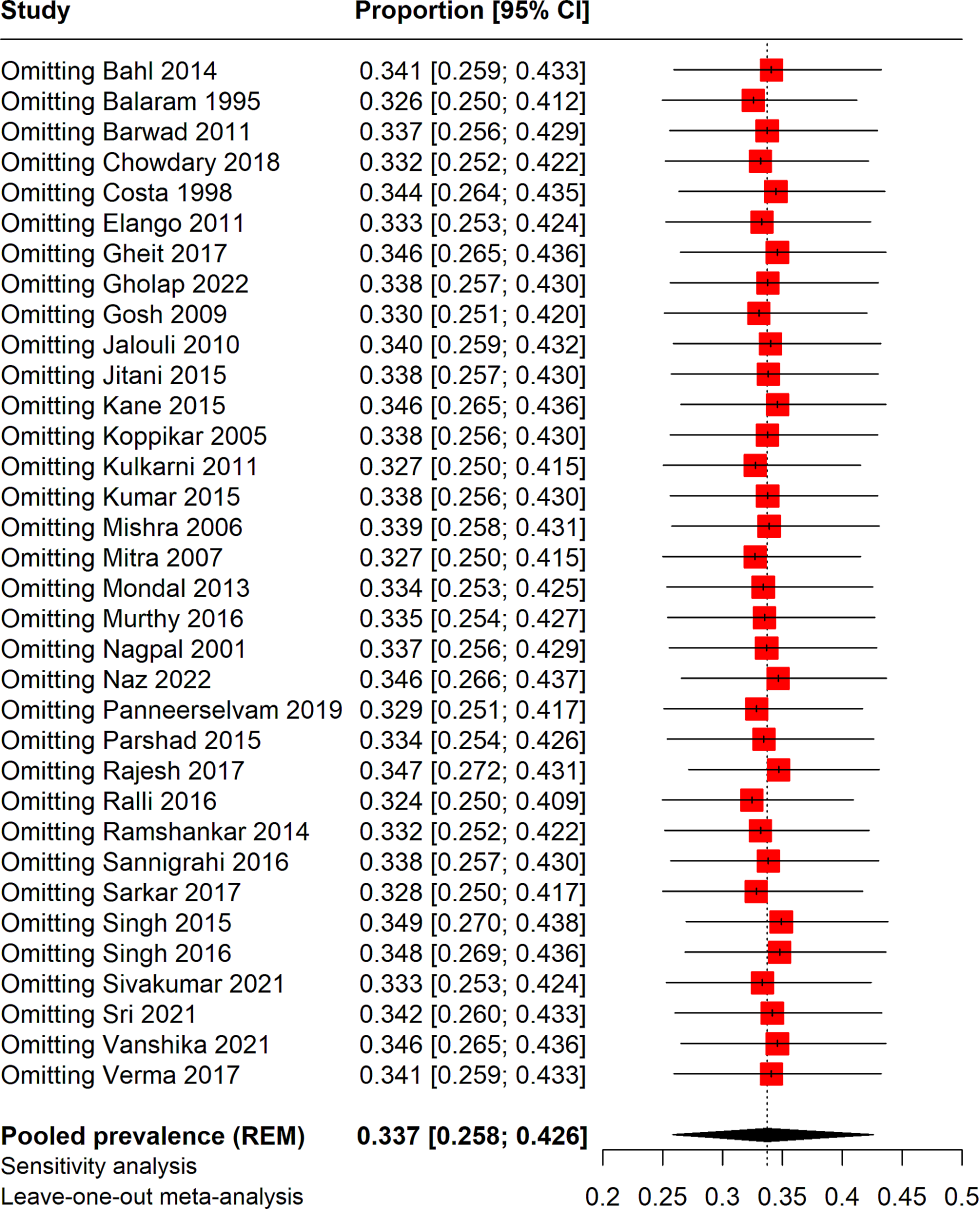
Sensitivity analysis of pooled prevalence

Meta-regression was performed to determine the effect of sample size on the pooled prevalence of HPV in HNC cases, as depicted in Fig. 4. However, the meta-regression analysis indicated that sample size was not significantly associated with the pooled prevalence results (*p* = 0.20). Additionally, a leave-one-out sensitivity analysis was conducted to identify individual studies that might affect the overall prevalence rate. This analysis revealed that the exclusion of no single study resulted in any significant changes to the overall pooled prevalence, as illustrated in Fig. 5.

### Publication bias

Publication bias was assessed using funnel plot asymmetry and Egger’s test, as shown in Figure S1. The results of our analysis did not provide evidence of publication bias (Egger’s test, *p* = 0.71).

## Discussion

In the best of our knowledge, this is the first meta-analysis to determine the prevalence of HPV among HNC patients in India. In our analysis, we found an overall prevalence of 33% for HPV among HNC patients. South India and the eastern part of India have shown slightly greater prevalence compared to the north and west parts of India. Meta-regression, sensitivity analysis couldn’t resolve the s0000ource of heterogeneity.

Previous systematic reviews have established evidence that infection with HPV is a distinct risk factor for developing HNCs [61, 62]. The risk associated with tonsil carcinoma is notably high and aligns with what one would anticipate from an infectious cause. Our findings indicate that HPV’s involvement in tonsil cancer is similar to the role of Epstein-Barr virus in nasopharyngeal cancer, suggesting that this is yet another instance of a virus-induced cancer in the pharyngeal lymphoid tissue [62].

The risk associated with the progression or death, along with the likelihood of developing a secondary primary HNOSCC, increases by 1% and 1.5% respectively for each additional year of smoking. This remains true even when accounting for the HPV tumor status and other important factors. Importantly, the risk of death doubles for patients who continue smoking during radiation therapy. The detection of HPV in HNCs is highly significant for prognosis and can influence the modification of treatment plans based on the HPV status [63]. Thus, the accurate identification of HPV as a contributing factor in HNCs is of paramount importance. Typically, a biopsy or cytological analysis from the primary tumor site or enlarged lymph nodes is crucial for an initial diagnosis. PCR or RT-PCR (Reverse Transcriptase-PCR) are commonly employed methods for detecting HPV in tumor tissues, particularly for identifying E6/E7 in fresh frozen samples. However, these methods are associated with high setup costs and longer turnaround times [64].

The presence of HPV in HNCs significantly alters the disease’s prognosis and therapeutic approach. HPV-positive HNCs typically present with a better response to standard treatment modalities like radiotherapy and chemotherapy, leading to an overall better prognosis compared to HPV-negative cases [65]. This is primarily attributed to the distinct biological behavior of HPV-positive tumors, which tend to be more sensitive to radiation and cytotoxic agents. Studies has indicated that individuals diagnosed with HPV-positive oropharyngeal cancer have higher survival rates and lower risks of recurrence [66].

Given these differences, there is an emerging consensus on tailoring treatment strategies based on HPV status. For instance, the concept of treatment de-escalation for HPV-positive HNCs is gaining traction. This approach involves reducing the intensity of standard treatments to minimize long-term side effects without compromising the efficacy of cancer control [67]. Clinical trials are currently exploring various de-escalation strategies, including reduced-dose radiotherapy, omission of chemotherapy, and the use of minimally invasive surgeries [68, 69]. However, it’s important to approach de-escalation cautiously, ensuring that patient selection is based on robust biomarkers and clinical criteria to avoid under-treatment.

The accurate determination of HPV status in HNCs is pivotal for both prognostic assessment and guiding treatment decisions. Polymerase Chain Reaction (PCR) and Reverse Transcriptase-PCR (RT-PCR) are the gold standards for detecting HPV, particularly E6/E7 mRNA, in tumor tissues [70–72]. These methods are highly sensitive and specific but are often hindered by high costs and longer turnaround times, posing challenges in resource-limited settings. Emerging diagnostic techniques, such as liquid biopsies and next-generation sequencing (NGS), are promising alternatives. Liquid biopsies, which detect circulating tumor DNA (ctDNA) in blood samples, offer a less invasive method for HPV detection and monitoring [73–75]. NGS, on the other hand, allows for the comprehensive analysis of HPV integration sites and co-mutations, providing a more detailed tumor profile [76]. These technologies not only improve the accessibility of HPV testing but also enhance our understanding of the tumor biology, which is crucial for personalized medicine.

The significant role of HPV in HNCs necessitates public health interventions, particularly in countries like India where the burden of these cancers is high. HPV vaccination, which has been successful in reducing the incidence of cervical cancer, presents a viable strategy to curb HPV-related HNCs. The expansion of HPV vaccination programs to include both girls and boys could significantly reduce the future burden of these cancers [77, 78]. It is crucial to integrate HPV vaccination into national immunization programs and to raise public awareness about its benefits. Furthermore, the compounded risk of tobacco use in HPV-positive HNC patients highlights the urgent need for effective tobacco cessation programs. Tobacco, being a well-established risk factor for HNCs, exacerbates the risk even in the context of HPV-positive cancers. Public health campaigns targeting tobacco cessation are not only essential for preventing HNCs but also for improving outcomes in patients with existing HPV-related cancers [79–81].

Our study acknowledges certain limitations that should be considered. Firstly, our analysis was restricted to articles published in the English language, potentially omitting relevant research published in other languages. Additionally, the geographic coverage of our data is not comprehensive; studies from all regions of India were not available, which may affect the generalizability of our findings. While subgroup analysis was conducted, it was not sufficient to completely address the sources of heterogeneity observed in the results. We were unable to perform subgroup analysis based on site-specific cancer prevalence due to the unavailability of reported data. Future studies should include detailed data on site-specific HNC and HPV to facilitate more comprehensive analyses. This heterogeneity could stem from various factors such as differences in study design, populations, or methodologies, which our subgroup analysis could not fully disentangle. The overall sample size of the included studies was relatively small. This limitation could impact the statistical power of our findings and might lead to less precise estimates. Given these constraints, we recommend that future research should include a broader range of languages and geographic areas, especially underrepresented regions of India. More extensive studies with larger sample sizes would also be beneficial to provide more robust and generalizable results, and to further explore the sources of heterogeneity observed in this study. The absence of publication bias in our findings further strengthens the reliability of these insights.

## Conclusion

This comprehensive study underscores the significant role of HPV in the epidemiology of HNCs in India. Through a detailed systematic review and meta-analysis, we determined that the pooled prevalence of HPV among HNC patients in India is 33%, revealing a substantial impact of this virus on such cancers. Notably, the study highlighted regional variations, with the highest prevalence in eastern India (47%) and lowest in the western regions (∼ 20%). This variability suggests the influence of regional factors in the prevalence and impact of HPV in HNCs. Overall, our study provides valuable data for healthcare professionals and policymakers, emphasizing the need for targeted interventions and policies to address the HPV-related burden in HNC patients in India, while also considering the regional disparities in prevalence and risk factors.

### Electronic supplementary material

Below is the link to the electronic supplementary material.

Supplementary Material 1

## Data Availability

All the data used in this review has been provided in the manuscript and supplementary files.
